# Homogenous Good Outcome in a Heterogeneous Group of Tumors: An Institutional Series of Outcomes of Superficial Soft Tissue Sarcomas

**DOI:** 10.1155/2015/325049

**Published:** 2015-11-08

**Authors:** Valerie Francescutti, Sartaj S. Sanghera, Richard T. Cheney, Austin Miller, Kilian Salerno, Rachel Burke, Joseph J. Skitzki, John M. Kane

**Affiliations:** ^1^Department of Surgical Oncology, Roswell Park Cancer Institute, Buffalo, NY 14263, USA; ^2^Department of Pathology and Laboratory Medicine, Roswell Park Cancer Institute, Buffalo, NY 14263, USA; ^3^Department of Biostatistics and Bioinformatics, Roswell Park Cancer Institute, Buffalo, NY 14263, USA; ^4^Department of Radiation Medicine, Roswell Park Cancer Institute, Buffalo, NY 14263, USA; ^5^Naval Medical Center Portsmouth, Portsmouth, VA 23708, USA

## Abstract

*Introduction*. Superficial soft tissue sarcomas (S-STS) are generally amenable to wide excision. We hypothesized that local recurrence (LR) should be low, even without radiation therapy (RT), and sought to examine the contribution of depth to LR and OS. *Methods*. Patients with S-STS were retrospectively reviewed. Demographics, tumor features, treatment received, and outcomes were analyzed. *Results*. 103 patients were identified. Median age was 55 years; 53% of patients were female. Tumor site was 39% in trunk, 38% in the lower extremity, 14% in the upper extremity, and 9% in other locations. The most common histology was 36% leiomyosarcoma. Median tumor size was 2.8 cm (range 0.2–14 cm). Sixty-six percent of tumors were of intermediate/high grade. RT was administered preoperatively in 6% of patients and postoperatively in 15% of patients. An R0 resection was accomplished in 92%. At a median follow-up of 34.2 months (range 2.3–176), 9 patients had a LR (8.7%). Tumor size and grade were not associated with LR. OS was not associated with any tumor or patient variables on univariate analysis. *Conclusions*. LR was low for S-STS, even with large or high grade tumors and selective use of RT. Surgical resection alone may be adequate therapy for most patients. Superficial location seems to supersede other factors imparting a good prognosis for this group of tumors.

## 1. Introduction

Sarcomas are a heterogeneous group of solid tumors accounting for 1% of adult malignancies, with an annual incidence of soft tissue sarcoma (STS) of approximately 11,000 cases in the United States [1]. The heterogeneity of these tumors is significant with at least 50 different histologic subtypes of sarcoma identified, all of which have distinct biologic behavior and response to various treatments [2]. Further heterogeneity exists in that primary STS can occur in a myriad of anatomic locations including extremities, trunk, retroperitoneum, and head and neck [3].

Surgery, radiation, and systemic therapies are important treatment modalities in the treatment of patients with sarcoma. The aim of the local therapies includes negative margin resection with surgery and selective use of pre- or postoperative use of radiation therapy (RT) to decrease the rate of local recurrence (LR). There have been several factors that have been shown to predict risk for LR, including tumor size, tumor depth, histologic grade, surgical margin status, and the use of RT [4–9].

Superficial STS (S-STS) are a distinct group characterized by location above the superficial fascia, a factor that influences the T-stage in the AJCC Sarcoma Staging System [10]. Superficial depth has been demonstrated in a number of studies to portend a good prognosis with respect to metastasis-free survival (MFS) [2, 3, 11–13]. Tumor depth in this group of sarcomas has been shown to be as predictive of behavior as size [11]. Retrospective series of S-STS have shown favorable prognosis, with overall low local recurrence (LR) rates and excellent overall survival (OS) rates [14–16]. In addition, many LRs are salvageable with further surgery and RT [17].

Although multimodality treatment including RT or chemotherapy may be appropriate in the management of select S-STS, the majority can be managed by surgical resection alone as resection with widely negative margins is technically feasible in a greater proportion of these cases than in deep STS [18]. Moreover, the timing of RT or chemotherapy with respect to surgery in S-STS is not as well defined as deep sarcomas and is generally the result of multidisciplinary discussion related to tumor histology and grade and the ability to achieve a resection with wide margins and pathologic features following resection.

The current study was undertaken to determine the outcomes of our series of patients with S-STS, including LR and OS rates. In addition, we sought to describe the use of therapies other than surgical resection such as RT and chemotherapy in the treatment of S-STS patients. For a more comprehensive evaluation of the management and outcomes of S-STS given the smaller numbers of patients in individual series, we also performed a comprehensive literature review of outcomes specific to S-STS to provide an overview of the management of this unique disease entity.

## 2. Methods

### 2.1. S-STS Patient Series

#### 2.1.1. Patients

Institute ethics approval was obtained prior to study initiation. Charts of consecutive patients with S-STS undergoing definitive treatment from 1 January 1993 to 1 March 2011 at Roswell Park Cancer Institute (RPCI) were retrospectively reviewed. Patients were included in the study if they had a biopsy-proven sarcoma, superficial to fascia, and underwent treatment with curative intent (stages I–III). All initial biopsies (core needle or incisional) completed at outside institutions were reviewed by RPCI pathologist. Preoperative imaging generally included CT or MRI of the primary site at the discretion of the surgeon. Patients were excluded if tumor histology was dermatofibrosarcoma protuberans or if stage IV disease was identified at the time of planned initial surgical treatment. At the time of patient evaluation and treatment planning, malignant fibrous histiocytoma (MFH) was a histologic entity. Although presently this term is no longer used, patient cases were not reclassified at the time of the retrospective review and analysis to reflect this, as this diagnosis was used to make clinical decisions at the time of patient treatment. Surgical margins were defined as negative (free of microscopic or macroscopic tumor), close (margin was less than 2 cm, due to anatomic constraints), or positive (R1).

#### 2.1.2. Data Collection

For each patient the following data were collected: age, sex, tumor location (upper or lower extremity, superficial trunk, and head and neck), tumor size, histology, grade, and stage. Data regarding surgical management, including details of resection, tissue defect closure, and margin status, were reviewed and collected. Use of multimodality therapy including RT and/or chemotherapy was assessed, and timing of each with respect to surgery was noted. Time to any event (LR and/or death) was determined from the date of diagnosis in months. All LRs were biopsy proven. For survival data, patients were censored at the date of last clinic visit. For date of death, this was obtained from patient charts or was determined from the RPCI death registry.

#### 2.1.3. Outcomes

The primary outcome was local recurrence (LR) rate. Secondary outcomes included local recurrence free survival (LRFS) and overall survival (OS) rates.

#### 2.1.4. Statistical Methods

To evaluate the effect of administration of RT, patients were classified as receiving or not receiving RT. In addition, the contribution of tumor size (≤5 cm and >5 cm) and tumor grade on OS, LR, and LRFS was evaluated. Between-group comparisons of the distributions of continuous and categorical variables were assessed using the Wilcoxon Rank-Sum Test and Fischer's exact test, respectively. Patients were censored at the date of death or last follow-up. Differences in the time to LR were displayed in Kaplan-Meier plots. *p* values less than 0.05 were considered statistically significant.

## 3. Results

### 3.1. S-STS Patient Series

A total of 103 consecutive patients were identified for review.  Table 1 includes demographic data for all patients, indicating a median age of 55 years, with an approximately equal sex distribution. The majority of S-STS was located in the lower extremity (37.9%) or trunk (38.8%) and was generally smaller in size (≤5 cm). Median tumor size was 3.0 cm (range 0.15–14.0 cm). Approximately one-half of tumors were identified as either leiomyosarcomas or MFH, with other histologies represented in varying numbers.

Considering surgical management of S-STS, 92.2% of patients (*n* = 95) underwent an R0 resection (Table 1). Approximately one-third of patients required either a skin graft or flap for closure of the surgical site. Widely negative margins (≥2 cm) were achieved in 75% of patients undergoing surgery. Close margins, considered as margins <2 cm but not positive, occurred in 20% of patients, with the majority of these having a close deep fascial margin.

Regarding multimodality therapy, a total of 8 patients received chemotherapy in the management of their disease; 5 of these patients had angiosarcoma. Of the patients that received chemotherapy preoperatively (*n* = 5), 3 had angiosarcoma, and 2 had “other” histologies. Of those receiving postoperative chemotherapy (*n* = 3), 2 had angiosarcoma, and 1 was noted to have lymph node metastases postoperatively. Concerning use of RT, 75% of patients received no RT. Of those patients receiving RT, the majority of RT was administered in the postoperative period (*n* = 16). Of these 16 patients, most had a close surgical margin, especially deep (fascial).

**Figure 1:**
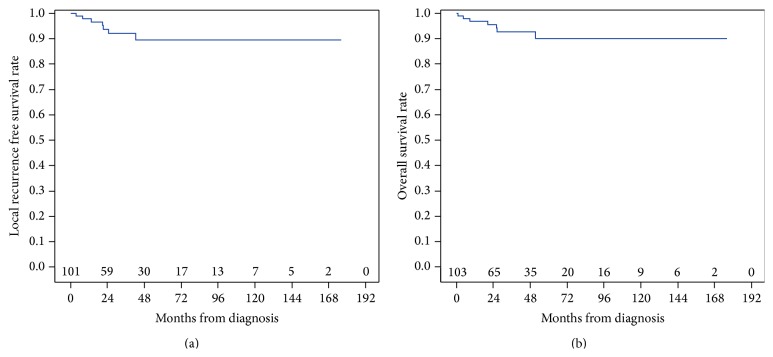
For all patients with S-STS, the local recurrence free survival (LRFS) rate was 93.2% (a), with median overall survival (OS) rate of 93.2% (b).

**Figure 2:**
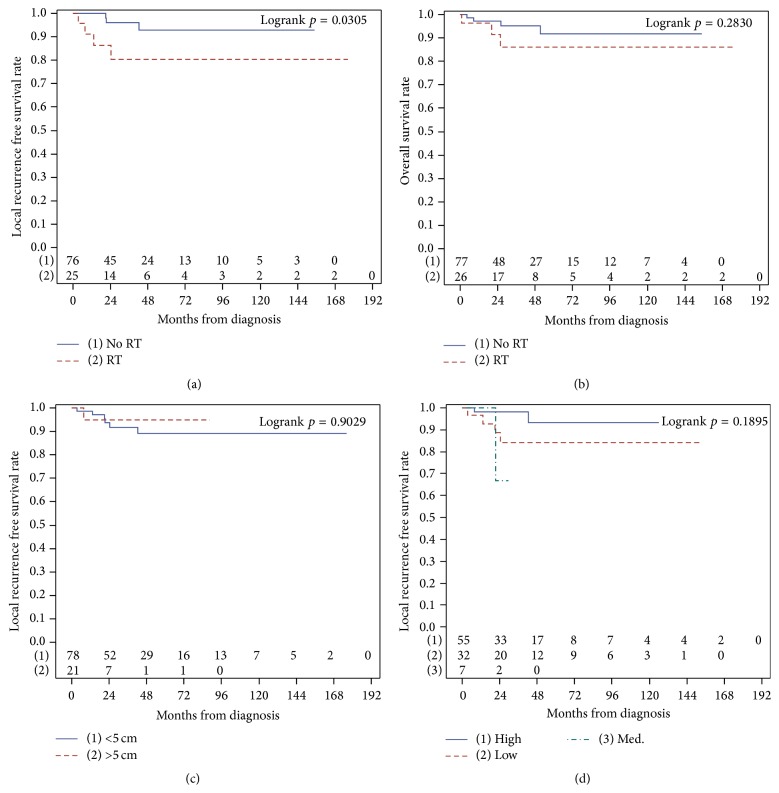
Dividing the cohort into those receiving radiation therapy (RT) either pre- or postoperatively, LRFS (a) was reduced in those patients undergoing RT as part of treatment (*p* = 0.03), whereas median OS (b) was not different between the two groups (*p* = 0.28). Both tumor size (c) and tumor grade (d) did not have an effect on recurrence free survival.

Median follow-up for this cohort of patients was 34.2 months (range 2.3–176.0 months; Table 2). A total of 6 patients developed distant metastases in the follow-up period, with 4 developing lung metastases. Two patients developed lymph node metastases following resection of the primary S-STS. The overall LR rate was 8.7%. The OS for this cohort was 93.2%. LRFS and OS for the entire cohort can be found in Figures 1(a) and 1(b), respectively. Considering patients undergoing RT, LRFS was significantly lower in those patients undergoing RT (*p* = 0.03, Figure 2(a)), but there was no effect on OS compared with those not undergoing RT (Figure 2(b)).

Figure 2(c) indicates no effect of tumor size on LRFS (*p* = 0.46) and similarly no effect of tumor grade on LRFS (*p* = 0.79, Figure 2(d)).

## 4. Discussion

Tumor location exclusively above the fascia has been shown in prior reports to confer a favorable prognosis in studied cohorts of S-STS. S-STS account for 20–30% of all soft tissue sarcomas and as such it is important to have a more thorough understanding of their biological behavior to make informed treatment decisions.

This is a single institution series of S-STS managed surgically, with decisions on a case-by-case basis for the use of RT and chemotherapy. This series includes a variety of tumor locations and histologies very similar to other published series of S-STS, with the majority being T1a sarcoma (5 cm or smaller). It has been suggested that most series of S-STS include smaller, low grade tumors, which are detected earlier, and that these factors could account for the better outcomes [15, 19, 20]. However, in our series, the majority of tumors were of intermediate to high grade. This is in keeping with the findings of Salas and colleagues, who found no correlation between grade and LRFS on multivariate analysis [16]. Regarding the issue of superficial versus deep location and earlier detection, Pisters and colleagues prospectively evaluated surgery alone with selective use of RT in T1 sarcomas of the extremity and superficial trunk and noted an overall LR rate of 29% (8/28) for deep and 6.7% (4/60) for superficial sarcomas. These two groups, which were very similar in terms of size and histology, still had discrepant LR rates [21]. This may be due to difficulty in achieving wide surgical margins in deep sarcomas as compared with those in superficial locations rather than a bias towards less aggressive smaller tumors that are detected earlier in the superficial group.

The approach to S-STS at our institution includes definitive surgical resection with wide margins. Wide margins were achieved in over 75% of patients. The LR rate in this series was low, at 8.7%, which compares favorably to other published series of S-STS [14–16, 19, 20]. Prior studies have indicated that the quality of surgical therapy is an independent prognostic factor for LRFS on multivariate analysis and this probably accounts for the favorable outcomes observed in our series [16, 20].

The local recurrence rate in our series was 8.7%. A total of 9 patients had a local recurrence. Of these 9 patients, 3 patients had wide margins (>/=2 cm), 2 had close margins (<2 cm), 2 had positive margins, and 2 had unknown margins. Four of the 9 patients with LR did not receive any RT. Both patients with positive margins received postoperative RT; one patient with close margins received postoperative RT. The second patient with close margins received RT postoperatively after resection of the local recurrence. Of those patients developing a LR, 6 patients had a single LR, while 3 had more than one LR (range 2-3). All LRs were managed with repeat surgical resection, some with the addition of RT. The overall low LR rate, in addition to the ability to successfully manage LR with surgical resection, is likely a reflection of more locally aggressive histology or tumor behavior in this small subset. In addition to surgery, consideration regarding the benefit of RT occurred on a case-by-case basis either by the treating physician or at the multidisciplinary team conference meeting. RT was used infrequently, in only 21.3% of patients in the pre- or postoperative period related to their initial resection. Chemotherapy was used most frequently in angiosarcoma patients, with a small number of other sarcoma patients receiving chemotherapy for treatment after the development of distant metastases.

Considering the effect of RT on OS and LRFS, it appears that those patients receiving RT had a poorer LRFS (*p* = 0.03), whereas OS was not affected. A total of 5 patients experiencing LR (55.6%) underwent RT treatment, 2 postoperatively after the initial resection and 3 after the LR. The poorer LRFS in the group receiving RT may be indicative of selective administration of RT in patients with known high-risk tumor biology or those in whom wide resection may be technically more difficult, with those more likely to develop a LR receiving RT. This is consistent with the findings of Coindre and colleagues, who noted a similar trend toward increased LR in the subset of patients to whom RT was administered because of a perceived high risk of recurrence based on the judgment of experienced clinicians [2].

Upon comprehensive review of the literature, only a small number of studies reported the outcomes of LR and OS in patients with S-STS separate from deep STS (Table 3). Overall, a total of 1024 cases in 6 series of S-STS have been documented, and results indicate a variable LR rate (8.0–24.8%). Of note, there is some variability in terms of S-STS included in each series, as two series included patients with DFSP, known to have a higher LR rate than other S-STS [15, 16]. The variable use of RT in these series, ranging from 8.5% to 52.8% of cases, indicates that no consensus exists on which patients may benefit most from this therapy. OS rates all fell within the same range for the various published series, although many had differing rates of histologic subtypes, tumor sizes, and proportion of patients receiving wide excision. Biau and colleagues evaluated the risk of LR in sarcoma based on several predictors in 1668 patients with localized STS of the extremity or trunk with a competition model. Their conclusions, similar to the practice at our institution, were the use of RT related to presentation status and surgical margins, with little influence of grade and tumor size [22].

These results taken together with our current series indicate that superficial anatomic location may supersede histology and size with respect to outcomes such as OS. One exception to this generalization is superficial angiosarcoma, where two larger series indicate a very different 5-year OS, between 34 and 45%, with very high LR rates (25–50%) [23, 24]. Although small numbers of angiosarcoma patients were included in most series, the rarity of this type of sarcoma did not affect OS rates, although contributing to the number of patients receiving chemotherapy.

The limitations of this study are related to its retrospective nature, including missing data, in particular related to overall tumor size when an excisional biopsy was completed at an outside facility. In addition, although this series involved low rates of RT use, information regarding surgical morbidities previously shown to be more frequent in STS such as surgical site infection and skin graft/flap failure was not collected.

Lastly, survival analysis may be limited by the fact that there were very few adverse outcomes. Only a univariate analysis of risk factors was performed, as a multivariate analysis was not possible due to the small number of events. Therefore the study was not adequately powered to accurately assess the impact of individual prognostic factors on survival.

Overall, the excellent outcomes for S-STS patients at our center highlight the importance of multidisciplinary management of such patients at specialized centers that can offer expertise through sarcoma pathology, surgical oncology, plastic surgery, radiation oncology, and medical oncology, for appropriate selection and timing of therapies for this group of patients.

## 5. Conclusion

Overall S-STS are well managed with surgical resection with wide margins as primary therapy. Selective use of RT in combination with appropriate surgical therapy has led to low LR rates, and these LRs are oftentimes managed adequately with repeat surgical resection. The treatment of sarcomas at centers experienced in multidisciplinary management through surgical resections and selective use of RT is integral to good outcome in this heterogeneous group of patients.

## Figures and Tables

**Table 1:** S-STS patient demographics, disease characteristics, and management.

	*n* = 103
Age, years	
Median (range)	55 (15–89)
Sex	
Male	48 (46.6%)
Tumor location	
Trunk	40 (38.8%)
Lower extremity	39 (37.9%)
Upper extremity	14 (13.6%)
Head and neck	8 (7.8%)
Vulva	2 (1.9%)
Tumor size	
≤5 cm	79 (76.6%)
>5 cm	21 (20.4%)
Unknown	3 (3.0%)
Histology	
Leiomyosarcoma	37 (35.9%)
MFH^1^	16 (15.5%)
Liposarcoma	8 (7.8%)
Pleomorphic sarcoma	8 (7.8%)
Myxofibrosarcoma	7 (6.8%)
Angiosarcoma	6 (5.8%)
Other^2^	21 (20.4%)
Grade	
Low	32 (31.0%)
Intermediate	7 (6.8%)
High	56 (54.4%)
Unknown	8 (7.8%)
Stage	
I	31 (30.0%)
II	49 (47.6%)
III	22 (21.4%)
Unknown	1 (1.0%)
Surgery, resection	
R0	95 (92.2%)
Wide (≥2 cm)	76 (80%)
Close (<2 cm)	19 (20%)
R1	6 (5.8%)
Unknown	2 (1.9%)
Surgery, closure of defect	
Skin graft	22 (21.4%)
Flap	14 (13.6%)
Radiation therapy	
Preoperative	6 (5.8%)
Postoperative	16 (15.5%)
None	77 (74.8%)
Other^3^	4 (3.9%)
Chemotherapy	
Preoperative	5 (4.9%)
Postoperative	3 (2.9%)
None	95 (92.2%)

^1^Malignant fibrous histiocytoma.

^2^Including epithelioid sarcoma, fibrosarcoma, synovial sarcoma, clear cell sarcoma, primitive neuroectodermal tumor (PNET), malignant peripheral nerve sheath tumor (MPNST), and not otherwise specified (NOS).

^3^Received after LR prior to excision.

**Table 2:** Outcome measures following treatment for S-STS.

Follow-up (months)	
Median (range)	34.2 (2.3–176.0)
Locoregional or distant metastasis site	
Lung	4 (3.9%)
Lymph node	2 (1.9%)
Liver	1 (1.0%)
Bone	1 (1.0%)
Local recurrence	9 (8.7%)
Overall survival	93.2%

**Table 3:** Literature review of treatment and outcomes for S-STS.

Series	*n*	Histologies	Location	Median size (range)	T-stage	Margin status	% receiving RT	Median follow-up (range)	LR rate	5-year OS
Rydholm et al., 1991 (Sweden) [19]	129	LMS, 18%	EX, 85%	4.3 cm (1–16 cm)	≤5 cm, 58%	Wide, 58.1%	8.5%	NR	24.8%	80%
		MFH, 46%	T, 15%		>5 cm, 38%	Close, 41.9%				
		LPS, 10%	H/N, 0%		Unknown, 4%					
		AS, 0%	Other, 0%							
		Other, 26%								

Brooks et al., 1998 (USA) [14]	215	LMS, 16%	EX, 100%	NR	<5 cm, 75%	R0, 91.6%	25%	45 months (2 d–151 months)	14%	79%
		MFH, 43%	T, 0%		≥5 cm, 25%	R1, 8.4%				
		LPS, 18%	H/N, 0%							
		AS, 3%	Other, 0%							
		Other, 20%								

Cany et al., 1999 (France) [15]	105	LMS, 20%	EX, 56.1%	3 cm (1–15 cm)	≤5 cm, 65.7%	Wide, 85.4%	52.8%	111.9 months (19.2–321.9 months)	10.5%	75%
		MFH, 39%	T, 27.7%		>5 cm, 34.3%	Close, 14.6%				
		LPS, 3%	H/N, 16.2%							
		AS, 5%	Other, 0%							
		Other^2^, 33%								

Salas et al., 2009 (France) [16]	367	LMS, 22.3%	EX, 55.0%	NR	≤5 cm, 76.3%	Wide, 76.3%	35.4%	74.2 months	23.4%	80.9%
		MFH^1^, 9.0%	T, 35.4%		>5 cm, 21%	Close, 21.2%				
		AS, 14.4%	H/N, 8.0%		Unknown, 2.7%	Unknown, 2.5%				
		Other^2^, 54.3%	Other, 1.6%							

Tsukushi et al., 2012 (Japan) [20]	105	LMS, 7.6%	EX, 73.3%	NR	≤5 cm, 45.7%	R0, 95.2%	4.8%	NR	8.0%	95.3%
		MFH, 24.8%	T, 26.7%		>5 cm, 54.3%	R1, 4.8%				
		LPS, 19.0%								
		Other, 48.6%								

Francescutti et al., 2015 (USA) [present study]	103	LMS, 35.9%	EX, 51.5%	3 cm (0.15–14 cm)	≤5 cm, 79.0%	Wide, 75.2%	21.3%	34.2 months (2.3–176.0 months)	8.7%	93.2%
		MFH, 15.5%	T, 38.8%		>5 cm, 21.0%	Close, 18.8%				
		LPS, 7.8%	H/N, 7.8%							
		AS, 5.8%	Other, 1.9%							
		Other, 35.0%								

Total	**1024**									

^1^Myxofibrosarcoma in this series.

^2^Dermatofibrosarcoma protuberans (DFSP) in this series.

LMS, leiomyosarcoma; MFH, malignant fibrous histiocytoma; LPS, liposarcoma; AS, angiosarcoma; EX, upper or lower extremity; T, superficial trunk; H/N, head and neck; NR, not reported.
